# Life Cycle Assessment of Prefabricated Geopolymeric Façade Cladding Panels Made from Large Fractions of Recycled Construction and Demolition Waste

**DOI:** 10.3390/ma13183931

**Published:** 2020-09-05

**Authors:** Davor Kvočka, Anja Lešek, Friderik Knez, Vilma Ducman, Matteo Panizza, Constantinos Tsoutis, Adriana Bernardi

**Affiliations:** 1 Slovenian National Building and Civil Engineering Institute (ZAG), Dimičeva ulica 12, 1000 Ljubljana, Slovenia; davorkvocka@gmail.com (D.K.); anja.lesek@zag.si (A.L.); vilma.ducman@zag.si (V.D.); 2 National Research Council–Institute of Condensed Matter Chemistry and Technologies for Energy (ICMATE), Corso Stati Uniti 4, 35127 Padua, Italy; matteo.panizza@icmate.cnr.it; 3 Proigmenes Erevnitikes & Diahiristikes Efarmoges (AMSolutions), Dim. Glinou 7, 14122 Irakleio, Greece; constantinos.tsoutis@amsolutions.gr; 4 National Research Council–Institute of Atmospheric Sciences and Climate (CNR-ISAC), Corso Stati Uniti 4, 35127 Padova, Italy; a.bernardi@isac.cnr.it

**Keywords:** life cycle assessment (LCA), construction and demolition waste (CDW), façade cladding panel, geopolymer, alkali-activated material (AAM)

## Abstract

The construction and demolition sector is one of the biggest consumers of natural resources in the world and consequently, one of the biggest waste producers worldwide. The proper management of construction and demolition waste (CDW) can provide major benefits for the construction and recycling industry. However, the recycling rate of CDW is relatively low, as there is still a lack of confidence in the quality of recycled CDW materials. Therefore, new research projects are looking for innovative solutions within recycling of CDW in order to overcome uncertainties currently associated with the use of construction products made from recycled or re-used CDW. In this paper, a “cradle-to-cradle” life cycle assessment (LCA) study has been conducted to investigate the environmental performance of the prefabricated geopolymeric façade cladding panels made from large fractions of CDW. The LCA results indicate that the majority of the environmental burden arises within the manufacturing stage; however, the environmental burden can be reduced with simple optimisation of the manufacturing process. Furthermore, the environmental impact of the prefabricated geopolymeric façade cladding panels is generally lower than the environmental burden associated with the façade cladding panels made from virgin materials.

## 1. Introduction

The construction and demolition sector is one of the biggest consumers of natural resources, which consumes 25% of virgin wood, 17% of fresh water and 40% of all raw materials (e.g., stone, gravel and sand) extracted worldwide, and is responsible for around 40% of all of global greenhouse gas emissions [1,2]. Consequently, it is also one of the biggest waste producers in the world, which annually generates around 35% of all global waste [3]. For example, the construction and demolition sector generates 40% of total urban waste in mainland China, around 30% of total solid waste in the USA and approximately 46% of the total waste in Europe [4,5].

Construction and demolition waste (CDW) can be defined as a solid waste that is generated during the construction, maintenance, demolition and deconstruction of buildings and civil works. CDW mainly consist of mineral components, such as crushed concrete, bricks, tiles and asphalt, as well as plastics, wood, metals, glass and cardboard [6]. Even though there is a high potential for recycling and re-use of CDW, there is still a lack of confidence in the quality of CDW recycled materials [7]. This is primarily a result of inadequate information on recycled or re-used CDW products, negative perception associated with these products, unexpectedly high cost of CDW materials when compared to virgin materials and considerably conflicting information on availability, durability, qualities and functionality of CDW materials and products [8].

Therefore, further research is needed in order to develop innovative materials and solutions that would aim at overcoming uncertainties that are currently associated with the use of construction products made from recycled or re-used CDW. One of research projects that are looking for innovative solutions within recycling of CDW is also the InnoWEE project (H2020 InnoWEE: Innovative pre-fabricated components including different waste construction materials reducing building energy and minimising environmental impacts). The main objective of the InnoWEE project is the development of new high-performing prefabricated geopolymeric façade panels made from large fractions of recycled CDW, which would have a low environmental impact, low embodied energy, low CO_2_ emissions and high thermal performance.

Geopolymers or alkali-activated materials (AAM) are an environmentally friendly and technically promising alternative to cement, concrete and ceramics. In order to produce geopolymeric products, two main components are needed: (i) SiO_2_ and Al_2_O_3_ in sufficient quantities and reactive form-glassy state, and (ii) alkaline activators in a solution (e.g., NaOH, KOH, Na-water glass, K-water glass). When these two components are mixed, first, dissolution and transport of aluminium and silicate components in the alkaline activators takes place, and then an aluminosilicate network is formed through poly-condensation of the Al and Si components. Many naturally occurring materials, such as thermally activated clay or natural pozzolan (e.g., volcanic ash), as well as industrial waste (e.g., fly ash, bottom ash, and various slags), can serve for these purposes [9].

Life cycle assessment (LCA) is a standardized technique that addresses the environmental impacts associated with different stages of a product’s life cycle, such as raw material extraction, material processing and manufacturing of the product, product use, repair and maintenance, and finally, product re-use, recycling or final disposal [10,11]. Even though façade represents a key factor in the overall energy efficiency of a building, a limited number of studies have employed LCA to evaluate the environmental performance of façade cladding panels or façade systems. The majority of available studies are focused on traditional virgin materials (e.g., such as glass, stone or aluminium), where studies address a single product or compare different types of solutions made from the same material.

For example, LCA has been employed to evaluate energy consumption and CO_2_ emissions of a glass curtain wall system [12], or to compare the environmental performance of different glass window materials [13]. Traverso and co-authors [14] have analysed a typical Sicilian marble and determined that tile manufacturing has higher values of embodied energy and environmental performance indicators when compared to the slabs. Gazi and co-authors [15] have compared six impact categories for marble tiles, with the highest impact being observed in terms of acidification and global warming. Ioannidou and co-authors [16] have compared the most commonly used stoned wall systems, where the analysis has shown that the use of thicker stone and the change in the structural system can present environmental benefits. On the other hand, there are no independent LCA studies focusing exclusively on aluminium panels [17].

There are only few studies that directly compared different façade systems of façade materials. Taborianski and Prado [18] have compared the entire life cycle CO2 emission of five different office building façades and found that the structural glazing façades with colourless glass had the highest CO_2_ emissions when compared to the ceramic brick façades and façades built using brick and covered with mortar. Han and co-authors [17] have evaluated the environmental performance of ceramic façade panels and compared it to three other façade materials (i.e., marble, glass and aluminium), with the ceramic panels generally being more environmentally friendly than the other three materials.

In the field of CDW management, LCA has been applied to investigate the performance of general CDW management strategies and CDW recycling plants, compare natural material to recycled material from CDW, analyse different end-of-life scenarios for buildings and evaluate electricity production from CDW [19]. In addition, LCA has also been applied to investigate the performance of alkali-activated materials, with the results suggesting that AAM products are generally more environmentally friendly than other technically competitive products, [20]. However, as far as the authors are aware, there is no LCA study that evaluates the entire life cycle of façade panels made from geopolymer or alkali-activated material. As no other material can be considered as an adequate substitute to evaluate an environmental impact of geopolymeric façade panels, a complete LCA study is needed.

Therefore, the objective of this study was to investigate the environmental performance of prefabricated geopolymeric façade cladding panels made from large fractions of recycled CDW. The environmental performance has been evaluated by means of a “cradle-to-cradle” LCA analysis, which has considered the entire life cycle of the geopolymeric façade panels. In addition, the environmental performance of the geopolymeric panels has been compared to the environmental performance of façade cladding panels made from virgin materials (i.e., marble, aluminium, glass and ceramic). This comparison has been based on a ”cradle-to-gate” LCA, as there was a lack of data to develop a full life cycle scenario for considered virgin materials.

## 2. Materials

The objective of the InnoWEE project is to use the geopolymeric technology to produce a system of panels able to incorporate high amounts of CDW. Two types of geopolymer mixtures have been developed within the InnoWEE project: (i) high-density geopolymer (HDG), which embeds approximately 50% by weight of inorganic CDW aggregates (from fired clay, mortar and concrete rubble), and (ii) wood geopolymer (WG), which incorporates at least 40% of CDW wood particles.

The geopolymer binder was based on commercial solid precursors, i.e., metakaolin (MK: M1000 from Imerys, France), ground granulated blast furnace slag (SL: LV 425 supplied by Minerali Industriali, Italy) and class F fly ash (FA: type EFA Füller HP supplied by BauMineral GmbH, Germany), with the latter being used in HDG only. The activation was achieved through aqueous solutions of either potassium silicate (HDG) or sodium silicate (WG), which were commercially available as well. Aggregates used in the HDG production came from inorganic non-hazardous CDW, classified either as 17.01.01 (concrete), 17.01.07 (mixtures of concrete, brick, tile and other ceramics) or 17.09.04 (mixed construction and demolition wastes), according to the European List of Wastes [21], which were ground to obtain recycled sands with a maximum nominal size of 2 mm. Organic particles for the WG production consisted in softwood from untreated construction waste (e.g., pallets, carpentry boards, etc.), which was shredded to obtain a suitable mix of flakes and chips, mostly less than 3 cm long with an aspect ratio lower than 8.

The development of the inorganic HDG mixture, carried out through an extensive experimental research, basically involved two phases. The first, described in detail in Panizza et al. [22], was devoted to identifying the main parameters affecting mechanical, physical and technological properties related to a possible industrial exploitation, whereas the second was focused on the optimisation of the mixture to comply with the requirements set by the pilot plant and by the prototype panels’ features, especially in terms of viscosity and setting time of the fresh paste, curing parameters and adequate strength and limited drying shrinkage of the hardened material. The final HDG formulation delivered the following properties: Brookfield viscosity (spindle n 5 at 1 rpm) in the range 120–150 Pa·s and setting time greater than 70 min at 23 °C, compressive strength of about 37 MPa and splitting tensile strength of about 3 MPa at 28 days of age, apparent density of 1.89 g/cm^3^, open porosity of about 30% and drying shrinkage lower than 0.25% after 6 months.

The WG is a material similar to cement-bonded particleboard, but with the wood aggregates kept together by a geopolymer binder able to set at ambient temperature. The WG for façade cladding panels, owing to the envisaged exterior application, embedded 40% by weight of wood particles, resulting in an apparent density of about 1.0 g/cm^3^ in dry conditions and comprised between 1.1 and 1.2 g/cm^3^ in environmental conditions (interior ambient). Strength and elastic modulus measured in 3-point bending were about 5.6 and 2.02 × 10^3^ MPa, respectively.

The prefabricated façade cladding panel has a square size with an edge 595 mm long. It was designed as a plate of HDG stiffened by a frame made by two horizontal main ribs (with cross-section 30 × 30 mm^2^) and four vertical thinner ribs with a tapered cross-section 10 to 15 mm thick. Three WG panels 7 mm thick were applied to the backside of the HDG plate, which were sized to fit the space among the ribs. Thanks to the design features and to the presence of the WG elements, which improves the resistance (especially the impact strength), the thickness of the HDG layer could be limited to 7 mm. Consequently, the cladding panel, with a mass of about 25 kg/m^2^, is generally lighter than similar items made with concrete or natural stone. Additionally, the HDG layer acts both as a protective shield from natural elements (e.g., wind, rain, snow, etc.) and as an aesthetic finishing.

**Figure 1:**
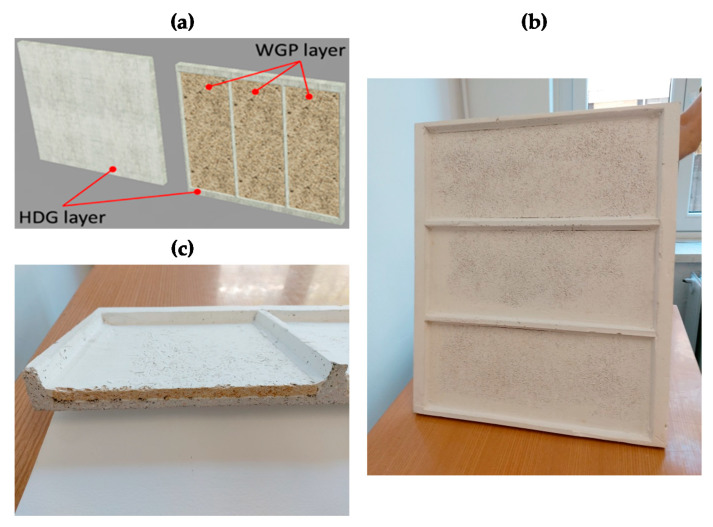
Sketch of the prefabricated geopolymeric façade cladding panel (**a**), developed panel (**b**) and panel cross-section (**c**) (the figure was produced by the authors).

The upscale production of the ventilated facade panels took place in the Technology Upscaling Pilot Plant (TUPP) in AMSolutions facilities. The upscale production includes the production of the high-density geopolymer (HDG) paste as well as the casting of the paste, the moulding, curing, demoulding, drying, painting, assembling of HDG with the WG in order to form the final panels and finally, packing of the final panels. For each one of the aforementioned production steps, the appropriate equipment and machinery was used, which includes mixers, electric pumps, electric motors, electromagnetic valves, storage silos, premixing buffers, weighting systems and many others. Eighty ventilated façade panels were produced in the TUPP in the framework of the InnoWEE project. The prefabricated geopolymeric façade cladding panel can be seen in Figure 1.

## 3. Application of the LCA Methodology

The LCA has been conducted in accordance with the principles and framework for LCA, which are defined in the international standard for LCA ISO 14040 and ISO 14044, and the European standard for Environmental Product Declarations (EPD) EN 15804. The LCA consists of four distinct phases: the goal and scope definition, the inventory analysis, the impact assessment and the interpretation of results [10,11]. The GaBi modelling software has been used to conduct the LCA study [23], which is one the most commonly used software programs in the field of LCA modelling [24].

### 3.1. Goal and Scope of the LCA

#### 3.1.1. Goal of the Study

The goal of the study is to investigate the environmental performance of the prefabricated geopolymeric façade cladding panels made from large fractions of CDW.

First, a “cradle-to-cradle” LCA will be conducted to identify the life cycle stage that contributes the most to the environmental footprint of the considered geopolymeric panels. 

Second, a detailed contribution analysis will be conducted for the life cycle stage that will be identified as the main contributor to the overall environmental burden, with the goal being to identify the main hotspot.

Third, a scenario analysis will be performed to test different life cycle configurations in order to reduce the impact of the processes with the largest environmental impact.

Finally, the environmental impacts of five different façade cladding materials will be compared based on a ”cradle-to-gate” LCA in order to determine the most environmentally friendly façade cladding material.

#### 3.1.2. Functional Unit

The functional unit for all types of LCA analyses considered in this study is 1 m^2^ of façade cladding panel.

#### 3.1.3. System Boundaries

**Figure 2:**
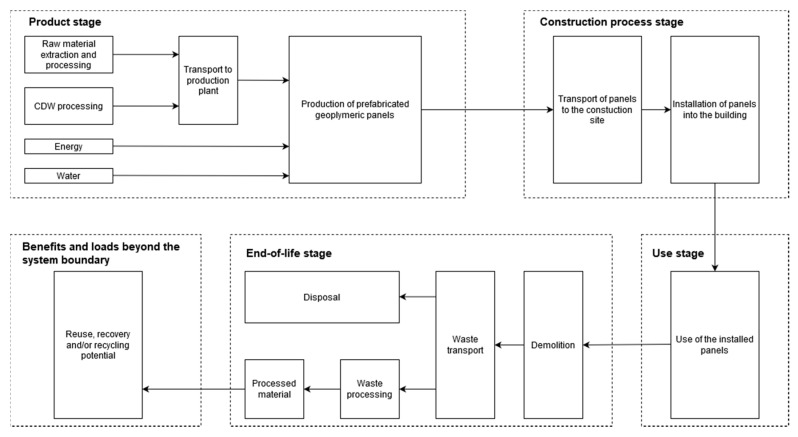
Schematic representation of the system boundaries (the figure was produced by the authors).

The “cradle-to-cradle” LCA considers the entire life cycle of the prefabricated geopolymeric panels: product stage, construction process stage, use stage and end-of-life stage. The schematic representation of the system boundaries is presented in Figure 2.

The product stage consists of (i) production of materials, including extraction of raw materials, (ii) transport of materials to the panel production plant and (iii) the production of the prefabricated geopolymeric panels, which includes mixing of the constituent materials, casting, curing, demoulding, drying, assembling and finally, packaging and storing of the produced prefabricated geopolymeric panels. The energy (e.g., mixing, casting, curing, transport inside the plant, etc.) and water (e.g., water for cleaning, etc.) consumption in the overall production process is also taken into consideration.

The construction process stage consists of transport of the panels to the construction site (i.e., module A4) and installation of the panels (i.e., module A5). A transport distance of 200 km has been selected and all supporting materials used within the installation process (e.g., fasteners, mortar, body anchors, etc.) have been included in the LCA model.

Once the panels are installed, there is generally no need for any maintenance or refurbishment, as well as operational energy or water use. Therefore, there are no impacts associated with the use stage of the prefabricated geopolymeric panels.

At the end-of-life stage, the geopolymer part of the panel (i.e., HDG) could be recycled and used as a replacement of virgin aggregate in roadbed, while non-recyclable parts of the panel would be disposed at landfill [25]. There are no impacts associated with the demolition of the panels (i.e., module C1), since the demolition is done manually. The non-recyclable parts have been transported to landfill (i.e., module C4), with the transport distance being set to 50 km. The geopolymer recycling rate has been set to 70%, which means that 70% of the geopolymer obtained after the demolition process has been transported to the recycling plant (i.e., module C3), while 30% has been transported to the landfill (i.e., module C4). The transport distance to the recycling plant (i.e., module C2) has been also set to 50 km. In the recycling plant, the geopolymer is crushed to the required particle size (i.e., module C3).

When the end-of-waste state is reached, it is assumed that the crushed geopolymer is going to be used in road base, which would avoid the use of virgin material for road construction. Therefore, the benefits and loads beyond the system boundary stage (i.e., module D), includes the impacts of the transport to the road construction site and the benefits (negative sign) due to the replacement of virgin material in the road construction. The transport distance to the road construction site has been set to 50 km, see Reference [25]. The secondary material (i.e., recycled geopolymer) does not have the same quality as the virgin material. Therefore, a value-correction factor has been applied in order to consider the difference in the material quality. A value of 0.5 has been applied for the value-correction factor, see Reference [25], which means that calculated benefits due to the replacement of virgin material are multiplied with the value-correction factor of 0.5.

#### 3.1.4. Allocation

As there are no by-products in the system, no allocation procedure has been performed.

### 3.2. Life Cycle Inventory (LCI)

The Ecoinvent cut-off system model has been used to model the recycled content (i.e., inorganic and organic CDW), which is compliant with the European standard EN 15804 [26]. The cut-off system model approach is based on the premise that primary (first) production of materials is always allocated to the primary user of a material. This means that secondary or recycled materials bear only the impacts of the recycling processes. For example, recycled inorganic CDW (e.g., brick) bears the impacts of CDW collection and the recycling process of CDW into a secondary material, while it is free of any burdens associated with the resource-extraction activities and processing required for the primary production of construction material (e.g., bricks).

Most of the considered raw material processing and secondary/auxiliary material production has been evaluated based on the LCI data given in the GaBi Professional database [23]. In addition, the delivery of all materials, the production of electricity and water supply have also been modelled based on the LCI data given in the GaBi Professional database. The GaBi Professional database considers only the emissions that are related to the operation of vehicles, power plants and wastewater treatment plants, whereas the emissions related to the construction of vehicles and/or the infrastructure are not included. For example, only burdens related to the production of fuel and its combustion in the truck’s engine are thus evaluated by the dataset in the GaBi Professional database [27]. The data on the electricity and water consumption has been provided by the operator of the panel pilot production plant.

Not all of the data for materials used in the production of panels are available in the existing databases. Therefore, upstream modelling for three input materials/production processes has been conducted: metakaolin, potassium silicate and specific CDW processing. Metakaolin is produced from kaolin, which is first crushed and dried and then calcined in a blast furnace. The upstream modelling of metakaolin thus considered raw material and energy (i.e., natural gas, electricity, biogas and sawdust) and is based on the specific plant production located in Italy. Potassium silicate is produced from a mixture of potassium hydroxide, silicon dioxide and water. The upstream modelling of potassium silicate thus considered raw material and energy (i.e., electricity and biogas) and is based on the specific plant production located in Italy. The CDW consists of a mixture of different building materials, such as brick, concrete, wood, steel, plaster, etc. The processing of CDW included sieving, crushing and milling of the material obtained from demolition, which consumed diesel, as a fuel for trucks, loaders and excavators, as well as electricity and water for mills and crushers. All these materials, processes and energy requirements have been considered, with CDW processing being based on specific processing located in Italy.

### 3.3. Life Cycle Impact Assessment (LCIA)

The environmental impact assessment has been calculated at the midpoint level with the CML 2001 (version January 2016) impact assessment method. The CML 2001 (version January 2016) is an impact assessment method that restricts quantitative modelling to early stages in the cause–effect chain to limit uncertainties, with results being grouped in midpoint categories according to common mechanisms (e.g., climate change) or commonly accepted groupings (e.g., ecotoxicity) [28].

The main principles behind the methodology are based on ISO 14040 and 14044 standards, and the characterisation factors are updated when new knowledge on substance level is available (e.g., the last update dates to January 2016) [23]. The results of the CML 2001 impact assessment method can be presented in terms of different impact potentials, which are summarised in Table 1.

The CML 2001 impact assessment method does not evaluate the primary energy demand (i.e., primary energy from renewable and non-renewable resources) and the total use of freshwater (i.e., freshwater consumption). Therefore, primary energy demand and total use of freshwater have been evaluated separately within the GaBi modelling software. The primary energy demand and the total use of freshwater potentials are summarised in Table 2.

**Figure 3:**
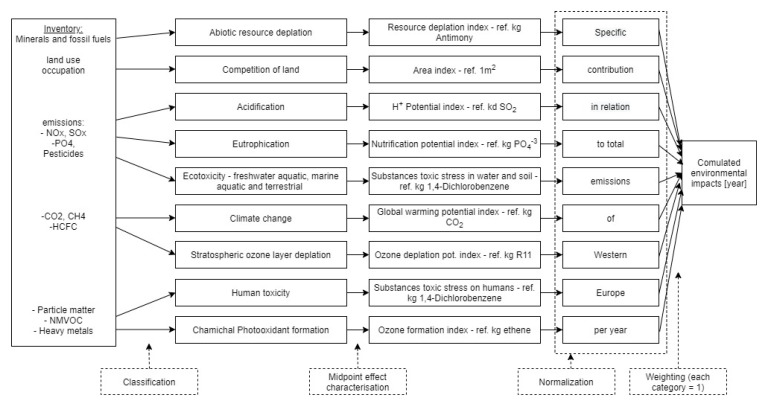
The general structure of the CML 2001 life cycle impact assessment method [28] (the figure was reproduced by the authors)**.**

The general structure of the CML 2001 life cycle impact assessment method is presented in Figure 3.

### 3.4. Life Cycle Interpretation

The interpretation phase of an LCA study generally consists of two types of interpretation steps: (i) procedural steps, which analyse data and results in relation to other sources of information, such as expert judgements and reports on similar products, and (ii) numerical steps, which analyse results without referencing to other sources of information [29]. The numerical approach of the interpretation phase generally consists of different types of analysis, such as contribution, scenario and comparative analysis.

The contribution analysis is used to decompose LCA results into number of constituent elements or contributions, which provide an overview of specific contributing factors. The contribution analysis is used in majority of LCA studies and is a self-evident method, which is the main reason why a clear exposition is often not written [29]. The results of a contribution analysis are usually expressed in percentages that add up to 100, which can be visualised with a pie chart or stacked bar diagram.

The scenario analysis is used to test various different configurations of the main modelling process and to observe the effect of these changes on the final model results [30]. The analysis can consist of comparing different case-specific downstream scenarios or comparing different variations of the same modelling process. The obtained results can be used to rank between different downstream scenarios or to find alternatives in the case of production hotspots.

The comparative analysis is used to simultaneously look at different product alternatives. It provides a very simple way to systematically look at model results of different scenarios in a way of a tabular list or a bar chart. However, the analysis has to be done with caution, since the user can be easily induced to make conclusions without any proper evaluation of the uncertainty of the analysis results [29].

## 4. Results and Discussion

### 4.1. Contribution Analysis

**Figure 4:**
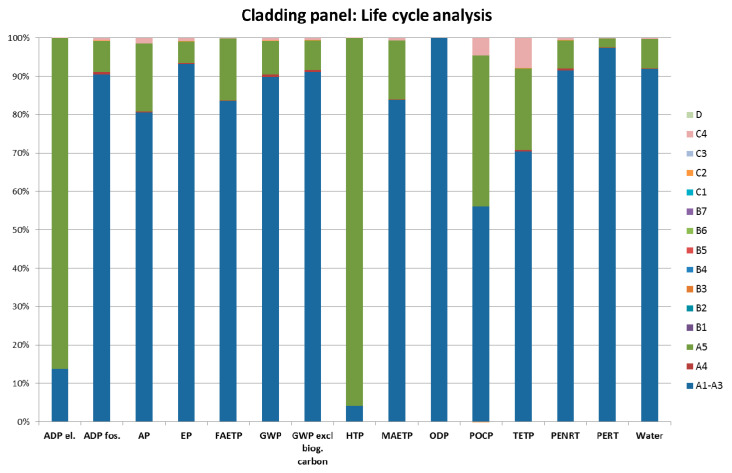
The environmental impacts associated with different life cycle stages of the prefabricated geopolymeric façade cladding panel (the figure was produced by the authors).

Figure 4 shows the environmental impacts associated with different life cycle stages of the prefabricated geopolymeric façade cladding panel. It can be seen from Figure 4 that the product stage (i.e., modules A1–A3) contributes the most to the environmental footprint of the considered prefabricated geopolymeric panel. The other life cycle stage that has a more significant environmental impact is the construction process stage (i.e., module A5) in terms of ADP el., HTP and POCP. Finally, Figure 4 shows that all other life cycle stages have minimal impact on the environmental performance of the prefabricated geopolymeric panels.

The results presented in Figure 4 have shown that the product stage (i.e., modules A1–A3) contributes the most to the environmental footprint of the prefabricated geopolymeric façade cladding panel. Therefore, a contribution analysis of the model results for the product stage of the considered prefabricated geopolymeric panels has been conducted in order to identify the main production hotspots. The hotspots can be defined as the processes that contribute the most to the total environmental footprint of the considered product. Therefore, if the main polluters within the production process are identified, more eco-friendly materials or processes can be considered as an alternative in order to reduce the environmental burden.

**Figure 5:**
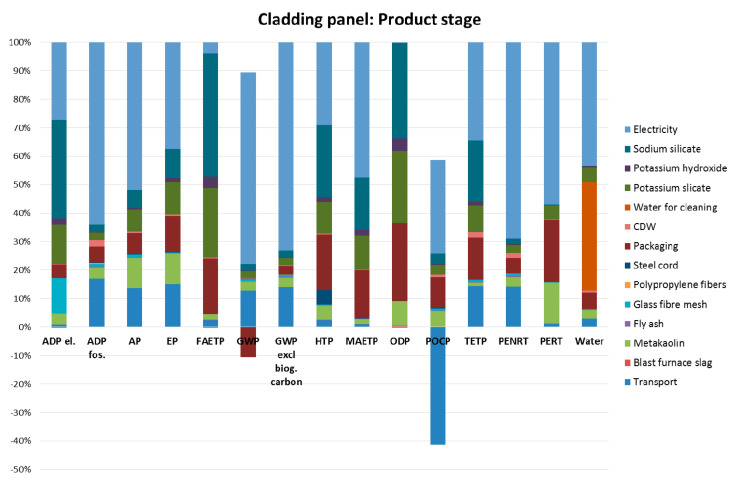
The contribution analysis of the model results for the product stage of the prefabricated geopolymeric façade cladding panel (the figure was produced by the authors).

Figure 5 shows the relative contributions of the constituent materials, supporting processes and energy and water requirements to the environmental footprint of the product stage of the prefabricated geopolymeric façade cladding panel. It can be seen from Figure 5 that the electricity requirements present the main production hotspot. For example, the electricity requirements represent 85% of the total impact in terms of the global warming potential (GWP), 52% of the total impact in terms of the acidification potential (AP) and 47% of the total impact in the terms of the marine aquatic ecotoxicity potential (MAETP).

This is mainly due to the fact that nearly 50% of the produced electricity in the EU comes from thermal power plants [31]. For example, the environmental impacts from thermal electricity based on lignite are roughly one to two orders of magnitude higher than those of nuclear power and renewable energy [32]. Therefore, the electricity requirements present negative impacts on climate, human health and the environment, mainly due to the greenhouse gas, particulate matter and other toxic emissions associated with the combustion of fossil fuels, such as coal or lignite.

The materials/processes that also contribute more significantly to the environmental performance of the product stage are the production of the sodium silicate, packaging and transport. All other materials/processes generally have minimal environmental impact, or they contribute more significantly only in terms of an individual impact category, such as metakaolin in terms of photochemical ozone potential creation (POCP) or glass fibre mesh in terms of the abiotic depletion (ADP el.).

Figure 5 also shows that some processes can have a positive environmental impact, such as packaging in terms of GWP and transport in terms of the photochemical ozone creation potential (POCP). The GWP is calculated by including the biogenic carbon, which can be found in biomaterials such as wood. The main component in the packaging process is the wooden pallet. The use of wood reduces the life cycle GHG emissions and temporarily stores the biogenic carbon in the anthroposphere [33]. This leads to the lower levels of embodied and operational carbon and thus to the positive impact in terms of GWP.

The photochemical ozone is generated by sunlight-initiated oxidation of volatile organic compounds (VOC) and carbon monoxide (CO) in the presence of nitrogen oxides (NOx) [34]. The volatile organic compounds react differently with different oxidants (e.g., ozone, NO_2_, etc.) and therefore can either have negative or positive effects on the ozone formation. The negative value of the transport in terms of the POCP is related to the separation of the NOx emissions in the NO_2_ and NO emissions, with NO and O_3_ (ozone) reacting to NO_2_ and O_2_ during the night time and thus leading to the reduction of the POCP [35].

### 4.2. Scenario Analysis

The results presented in Figure 5 have shown that the electricity requirements within the product stage contribute the most to the environmental footprint of the considered prefabricated geopolymeric façade cladding panels. The vast majority of the electricity consumed within the product stage is used to generate heat for curing of panels and heating of cleaning water. Therefore, the potential reduction of the electricity consumption could be achieved by considering a more environmentally friendly source of energy that is used for generation of heat.

**Figure 6:**
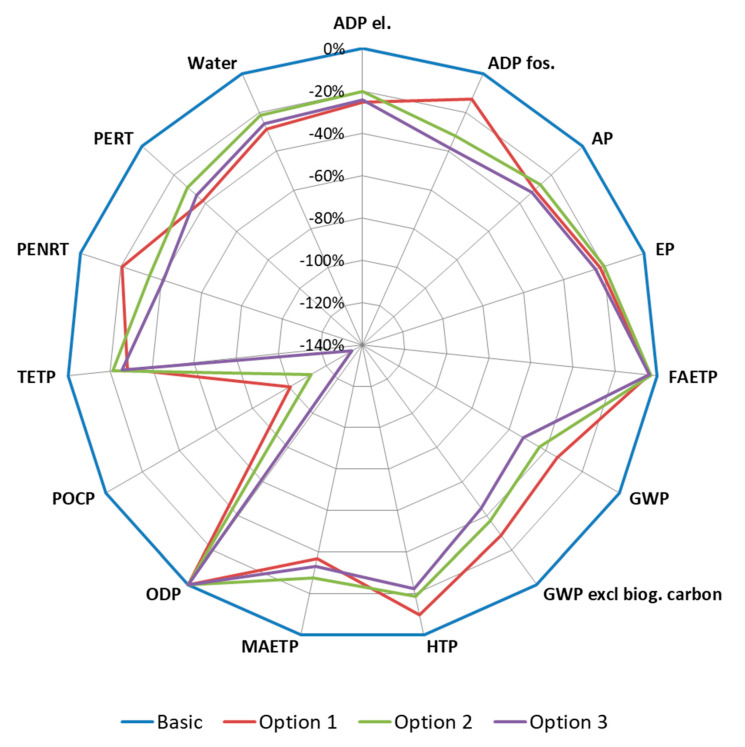
The decrease in the environmental burden for different heat generation scenarios when compared to the basic panel production process (the figure was produced by the authors).

Three different heat generation scenarios have been considered: (i) Option 1, where heat is generated from natural gas, (ii) Option 2, where heat is generated by using a heat pump and (iii) Option 3, where heat is generated by using solar panels. Figure 6 shows the reduction in the environmental burden associated with the product stage of the considered heat generation scenarios when compared to the basic prefabricated geopolymeric panel production process.

It can be seen from Figure 6 that all three scenarios lead to the reduction in the environmental burden, with the most beneficial scenario being Option 3 (i.e., heath generation by using solar panels). Namely, Option 3 leads to the largest or second-largest reduction in the environmental burden in terms of all considered impact categories. Option 2 (i.e., heath generation by using heat pump) is slightly better than Option 1 (i.e., heath generation from natural gas). This is mainly due to the better performance in terms of primary energy demand from non-renewable resources (PENRT) and depletion of abiotic resources (ADP fos.), as well as in terms of global warming potential (GWP) and human toxicity potential (HTP).

### 4.3. Comparative Analysis

The environmental performance of the prefabricated geopolymeric façade cladding panels has been compared to the environmental performance of façade cladding panels made from virgin materials, i.e., marble, aluminium, glass and ceramic. The data on environmental performance of cladding panels made from virgin materials has been obtained from Reference [17], with the data being based on the industrial manufacturing processes and surveys. The functional unit for the purposes of the comparative analysis is 1 m^2^ of the façade cladding panel. The comparative analysis has been conducted only for the product stage (i.e., modules A1–A3) of the considered façade cladding panels. This is due to the lack of data to develop a full life cycle scenario for considered virgin materials. As mentioned, the results suggest that the product stage generally contributes the most to the environmental footprint (see Figure 4). Therefore, a comparative analysis of the considered cladding materials based only on the “cradle-to-gate” LCA results should be indicative enough to evaluate the performance of the considered panels with relatively high confidence in the analysis results.

**Figure 7:**
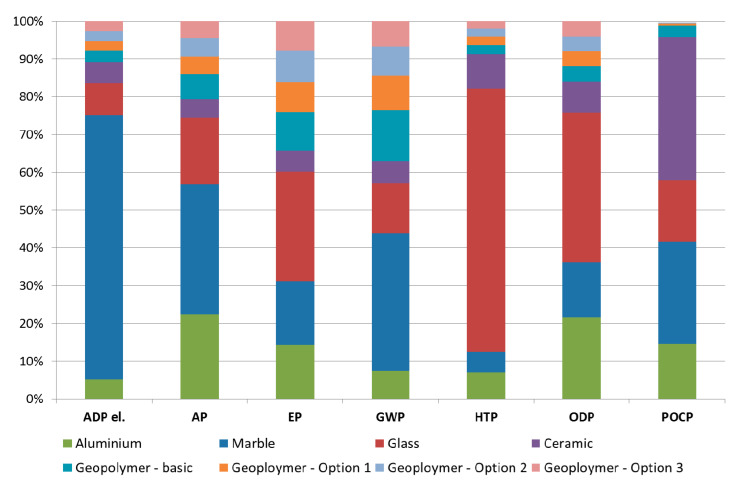
The comparison of the environmental performance of different façade cladding materials (the figure was produced by the authors).

Figure 7 shows the comparison of the environmental performance of the considered façade cladding materials. It can be seen in Figure 7 that marble and aluminium are the least environmentally friendly materials, since they have the largest or second-largest impact in terms of all seven considered impact categories. This is related to the high environmental burden associated with the raw material extraction (e.g., quarrying and mining, etc.) and manufacturing process (e.g., cutting, forging and pressing, etc.) of the marble and aluminium panel production [17]. The environmental performance of glass is generally better when compared to marble and aluminium, but on average still worse than ceramic and geopolymer. The ceramic and geopolymer generally have the lowest environmental impact, although the ceramic poses greater environmental burden in terms of HTP when compared to glass, and the geopolymer in terms of GWP when compared to glass and ceramic.

In terms of AP, EP and POCP impact categories, the geopolymer falls behind ceramic, while in terms of the GWP impact category, it falls behind ceramic and glass. This is mainly due to the high environmental burden associated with the electricity requirements in the product stage of the prefabricated geopolymeric façade cladding panel. However, when an alternative heat generation scenario is considered (e.g., Geopolymer—Option 1, Geopolymer—Option 2 and Geopolymer—Option 3), the geopolymer performs the best in all seven considered impact categories. All in all, the geopolymer can be considered as an environmentally friendly cladding material. Furthermore, with relatively simple modification of the production process, the prefabricated geopolymeric cladding panels generally have a noticeably lower environmental impact than cladding panels made from virgin material.

## 5. Conclusions

In this paper, we have investigated the environmental performance of the prefabricated geopolymeric façade cladding panels made from large fractions of construction and demolition waste. The study included: (i) contribution analysis of the product’s life cycle and identification of production hotspots, (ii) evaluation of alternative production process scenarios and (iii) a comparative analysis between geopolymeric façade cladding panels and façade cladding panels made from virgin materials, i.e., marble, aluminium, glass and ceramic.

The “cradle-to-cradle” LCA results have shown that the product stage (i.e., modules A1–A3) contributes the most to the environmental footprint of the considered prefabricated geopolymeric panels. The contribution analysis has highlighted that the electricity requirements present the main production hotspot and thus act as the main contributor to the overall environmental footprint of the considered geopolymeric façade panels.

Within the product stage, the majority of electricity is consumed to generate heat. Therefore, different production scenarios have been considered, where the heat has been generated from an alternative source of energy (i.e., natural gas, heat pump and solar panels). The scenario analysis has shown that the environmental burden has decreased in all three alternative scenarios, with the environmental impact in terms of an individual impact category decreasing on average between 20% and 40%. 

The comparative analysis based on the “cradle-to-gate” LCA suggests that the prefabricated geopolymeric façade cladding panels can be considered as an environmentally friendly construction product. Furthermore, when an alternative heat generation scenario is considered, the environmental impact of the panels made from geopolymer is up to 100% lower in terms of an individual impact category when compared to the panels made from virgin materials (particularly to marble, glass and aluminium).

It has been known that the use of waste material in the development of geopolymer or AAM can significantly contribute to the reduction of the CO_2_ footprint when these materials are compared to cement, concrete or ceramic [36]. The results presented in this paper have now also shown that not only is the material itself more environmentally friendly, but also that the final construction products made from geopolymer (e.g., façade panels) are generally more environmentally friendly than other technically competitive products. Furthermore, as the panels have been developed in order to allow for simple deconstruction and recycling, there is a lot of potential to develop new products that would incorporate large portions of recycled geopolymer and thus further minimise the environmental burden. 

Nonetheless, more research is needed that will further investigate the long-term technical performance, durability and economic feasibility of construction products made from recycled or re-used CDW. Such studies are needed in order to compile a pool of data that would show that construction products made from recycled or re-used CDW are as reliable as traditional construction products made from virgin materials. Only then would it be possible to fully exploit the environmental benefits of construction products made from recycled or re-used CDW as the main component in the promotion strategies to increase the social acceptability of these products. Namely, the wider social acceptance of concepts of sustainability and circular economy cannot be achieved without the participation of end-consumers that are willing to use the construction products made from recycled or re-used CDW.

## Figures and Tables

**Table 1:** CML 2001 midpoint impact categories.

Impact Category	Abbreviation	Unit
Global warming potential	GWP	(kg CO_2_ eq.)
Global warming potential excluding biogenic carbon	GWP excl. biog. carbon	(kg CO_2_ eq.)
Acidification potential	AP	(kg SO_2_ eq.)
Eutrophication potential	EP	(kg PO_4_^−3^ eq.)
Human toxicity potential	HTP	(kg DCB eq.)
Ozone layer depletion potential	ODP	(kg R11 eq.)
Photochemical ozone potential creation	POCP	(kg Ethene eq.)
Freshwater aquatic ecotoxicity potential	FAETP	(kg DCB eq.)
Marine aquatic ecotoxicity potential	MAETP	(kg DCB eq.)
Terrestrial ecotoxicity potential	FAETP	(kg DCB eq.)
Abiotic depletion (elements)	ADP el.	(kg Sb eq.)
Abiotic depletion (fossil)	ADP fos.	(MJ)

**Table 2:** The primary energy demand and the total use of freshwater potentials.

Impact Category	Abbreviation	Unit
Primary energy from non-renewable resources	PENRT	(MJ)
Primary energy from renewable resources	PERT	(MJ)
Freshwater consumption	WATER	(kg)
